# Publisher Correction: Promoter-proximal RNA polymerase II termination regulates transcription during human cell type transition

**DOI:** 10.1038/s41594-025-01521-9

**Published:** 2025-03-05

**Authors:** Kseniia Lysakovskaia, Arjun Devadas, Björn Schwalb, Michael Lidschreiber, Patrick Cramer

**Affiliations:** Department of Molecular Biology, Max Planck Institute for Multidisciplinary Sciences, Göttingen, Germany

**Keywords:** Transcription, Gene expression analysis

Correction to: *Nature Structural & Molecular Biology* 10.1038/s41594-025-01486-9, published online 11 February 2025.

In the version of the article initially published, the iMac II data (purple) in Fig. 3d were incorrect and have now been amended, as seen in Fig. 1. Additionally, the units shown for TT-Seq were “(0–292)” and have been corrected to “(0–400)”. The iMac I data (yellow) in Fig. 3d has been reformatted with thicker lines to promote visibility. These corrections have been made to the HTML and PDF versions of the article.

**Fig. 1:**
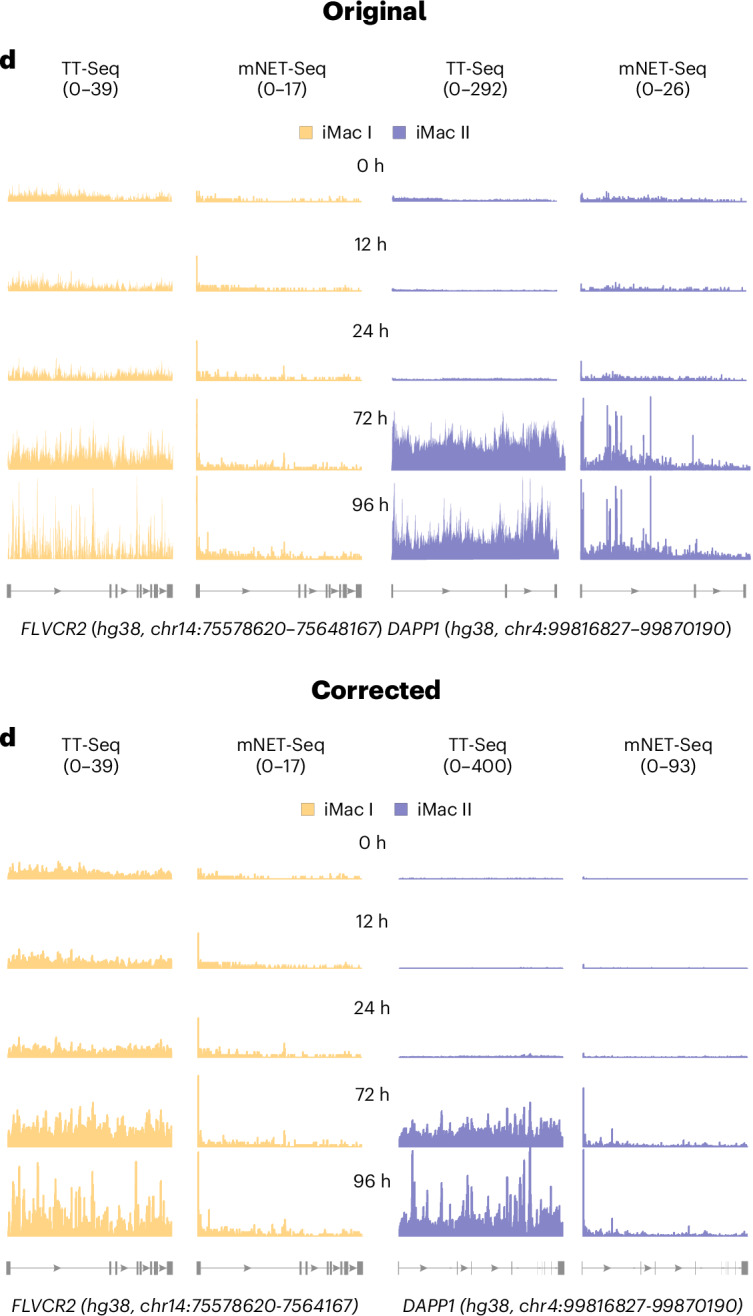
Original and corrected Fig. 3d.

