# Supplementary material for: Promoter-proximal RNA polymerase II termination regulates transcription during human cell type transition
Source: Nat Struct Mol Biol. 2025 Feb 11;32(6):995–1005. doi: 10.1038/s41594-025-01486-9 (PMC12170340; doi:10.1038/s41594-025-01486-9)
Supplement: Supplementary file 1 — Supplementary Tables 1–3. [file 41594_2025_1486_MOESM1_ESM.pdf]

nature structural & molecular biology

Supplementary information https://doi.org/10.1038/s41594-025-01486-9

Promoter-proximal RNA polymerase II termination regulates transcription during human cell type transition

In the format provided by the authors and unedited

Supplementary Table 1. Oligonucleotide sequences used in the RT-qPCR analysis of cell stage-specific marker expression during transdifferentiation

Designation	Source or reference	Sequence
IGJ forward	Rapino et al.[1]	TGTTCATGTGAAAGCCCAAG
IGJ reverse	Rapino et al.[1]	TCGGATGTTTCTCTCCACAA
VPREB3 forward	Rapino et al.[1]	GGGGACCTTCCTGTCAGTTT
VPREB3 reverse	Rapino et al.[1]	ACCGTAGTCCCTGATGGTGA
CD14 forward	Rapino et al.[1]	GATTACATAAACTGTCAGAGGC
CD14 reverse	Rapino et al.[1]	TCCATGGTCGATAAGTCTTC
FCGR1B forward	Rapino et al.[1]	CCTTGAGGTGTCATGCGTG
FCGR1B reverse	Rapino et al.[1]	AAGGCTTTGCCATTTCGATAGT
ITGAM forward	Rapino et al.[1]	GGGGTCTCCACTAAATATCTC
ITGAM reverse	Rapino et al.[1]	CTGACCTGATATTGATGCTG
GAPDH forward	Choi et al.[2]	TCTCTGCTCCTCCTGTTCGAC
GAPDH reverse	Choi et al.[2]	GGCGCCCAATACGACCAAAT

Supplementary Table 2. Reactome pathway analysis with STRING[3] of the iMac I gene set upregulated during transdifferentiation

Term description	Observed gene count	Background gene count	Strength	FDR
Neutrophil degranulation	25	473	0.74	2.24e-08
Innate Immune System	33	1025	0.53	1.41e-06
Immune System	45	1956	0.38	1.88e-05

Supplementary Table 3. Reactome pathway analysis with STRING[3] of the iMac II gene set upregulated during transdifferentiation

Term description	Observed gene count	Background gene count	Strength	FDR
Immune System	74	1956	0.55	3.11e-19
Neutrophil degranulation	31	473	0.79	2.92e-12
Cytokine Signaling in Immune system	32	681	0.64	3.66e-09
Innate Immune System	39	1025	0.55	5.07e-09
Signaling by Interleukins	24	440	0.71	7.41e-08
Interleukin-4 and Interleukin-13 signaling	13	107	1.05	1.76e-07
Interleukin-10 signaling	9	45	1.27	1.58e-06
Immunoregulatory interactions between a Lymphoid and a non-Lymphoid cell	11	129	0.9	8.29e-05
Caspase activation via Death Receptors in the presence of ligand	5	16	1.46	0.00055
MyD88 deficiency (TLR2/4)	4	10	1.57	0.0025
IRAK4 deficiency (TLR2/4)	4	11	1.53	0.0030
Cell surface interactions at the vascular wall	9	138	0.78	0.0047
Adaptive Immune System	21	743	0.42	0.0105
Regulation of TLR by endogenous ligand	4	19	1.29	0.0135
TRAIL signaling	3	8	1.54	0.0248
TRIF-mediated programmed cell death	3	9	1.49	0.0277

Toll-like Receptor Cascades	8	152	0.69	0.0345

Supplementary References

1. Rapino, F. et al. C/EBPalpha induces highly efficient macrophage transdifferentiation of B lymphoma and leukemia cell lines and impairs their tumorigenicity. *Cell Rep* **3**, 1153-63 (2013).
2. Choi, J. et al. Evidence for additive and synergistic action of mammalian enhancers during cell fate determination. *Elife* **10**(2021).
3. Szklarczyk, D. et al. STRING v11: protein-protein association networks with increased coverage, supporting functional discovery in genome-wide experimental datasets. *Nucleic Acids Res* **47**, D607-D613 (2019).
