# Supplementary material for: Promoter-proximal RNA polymerase II termination regulates transcription during human cell type transition
Source: Nat Struct Mol Biol. 2025 Feb 11;32(6):995–1005. doi: 10.1038/s41594-025-01486-9 (PMC12170340; doi:10.1038/s41594-025-01486-9)
Supplement: Supplementary file 2 — Reporting Summary [file 41594_2025_1486_MOESM2_ESM.pdf]

Corresponding author(s): Michael Lidschreiber
Last updated by author(s): Dec 8, 2024

Reporting Summary

Nature Portfolio wishes to improve the reproducibility of the work that we publish. This form provides structure for consistency and transparency in reporting. For further information on Nature Portfolio policies, see our Editorial Policies and the Editorial Policy Checklist.

Statistics

For all statistical analyses, confirm that the following items are present in the figure legend, table legend, main text, or Methods section.

n/a	Confirmed	
☐	☒	The exact sample size (*n*) for each experimental group/condition, given as a discrete number and unit of measurement
☐	☒	A statement on whether measurements were taken from distinct samples or whether the same sample was measured repeatedly
☐	☒	The statistical test(s) used AND whether they are one- or two-sided<br>*Only common tests should be described solely by name; describe more complex techniques in the Methods section.*
☒	☐	A description of all covariates tested
☐	☒	A description of any assumptions or corrections, such as tests of normality and adjustment for multiple comparisons
☐	☒	A full description of the statistical parameters including central tendency (e.g. means) or other basic estimates (e.g. regression coefficient) AND variation (e.g. standard deviation) or associated estimates of uncertainty (e.g. confidence intervals)
☐	☒	For null hypothesis testing, the test statistic (e.g. *F*, *t*, *r*) with confidence intervals, effect sizes, degrees of freedom and *P* value noted<br>*Give P values as exact values whenever suitable.*
☒	☐	For Bayesian analysis, information on the choice of priors and Markov chain Monte Carlo settings
☒	☐	For hierarchical and complex designs, identification of the appropriate level for tests and full reporting of outcomes
☐	☒	Estimates of effect sizes (e.g. Cohen's *d*, Pearson's *r*), indicating how they were calculated

Our web collection on statistics for biologists contains articles on many of the points above.

Software and code

Policy information about availability of computer code

Data collection	Sequencing data were collected using Illumina NextSeq 550.
Data analysis	Salmon v1.3.0; FASTQC v0.11.9; STAR v2.7.5a, Bowtie2 v2.3.4.1, Samtools v1.6, CutAdapt v2.3, R v4.2.0; DESeq2 v1.46.0, DAVID v2021q4; STRING db v11.5. Data analysis scripts: https://doi.org/10.5281/zenodo.14361017.

For manuscripts utilizing custom algorithms or software that are central to the research but not yet described in published literature, software must be made available to editors and reviewers. We strongly encourage code deposition in a community repository (e.g. GitHub). See the Nature Portfolio guidelines for submitting code & software for further information.

Data

Policy information about availability of data

All manuscripts must include a data availability statement. This statement should provide the following information, where applicable:

- Accession codes, unique identifiers, or web links for publicly available datasets
- A description of any restrictions on data availability
- For clinical datasets or third party data, please ensure that the statement adheres to our policy

Next-generation sequencing datasets generated in this study are available for download from GEO: GSE235181. Published TT-seq data used in this study is available for download from GEO: GSE131620.

Research involving human participants, their data, or biological material

Policy information about studies with human participants or human data. See also policy information about sex, gender (identity/presentation), and sexual orientation and race, ethnicity and racism.

Reporting on sex and gender	Not applicable
Reporting on race, ethnicity, or other socially relevant groupings	Not applicable
Population characteristics	Not applicable
Recruitment	Not applicable
Ethics oversight	Not applicable

Note that full information on the approval of the study protocol must also be provided in the manuscript.

Field-specific reporting

Please select the one below that is the best fit for your research. If you are not sure, read the appropriate sections before making your selection.

☒ Life sciences ☐ Behavioural & social sciences ☐ Ecological, evolutionary & environmental sciences

For a reference copy of the document with all sections, see nature.com/documents/nr-reporting-summary-flat.pdf

Life sciences study design

All studies must disclose on these points even when the disclosure is negative.

Sample size	All experiments were performed in two independent biological replicates. No statistical methods were used to pre-determine sample sizes, but our sample sizes are similar to those reported in previous publications by us (e.g., Choi et al., eLife 2021) and others (e.g., Shao et al., Nature Genetics 2017; Nojima et al., Cell, 2015). These sample sizes were chosen to generate data with sufficient depth and to robustly assess differences between time points.
Data exclusions	No data were excluded. Outliers were not drawn in the boxplots for clearer visualization.
Replication	All experiments involving genome-wide sequencing techniques were performed in two independent biological replicates. Western blotting and RT-qPCR experiments were independently repeated two times. All findings described in the manuscript were confirmed in all individual replicates.
Randomization	Cells were randomly seeded into the plates for the different time points of transdifferentiation for RT-qPCR, mNET-seq, ChIP- seq and ChIP-nexus experiments. Cells were randomly seeded into the plates for the different time points of DMSO and triptolide treatments for ChIP-nexus and Western blotting experiments.
Blinding	Blinding is not required. All samples were analysed using the same scripts and pipelines without any intervention by the investigator. Results were therefore directly related to the data and not influenced by any potential expectations of the researchers.

Reporting for specific materials, systems and methods

We require information from authors about some types of materials, experimental systems and methods used in many studies. Here, indicate whether each material, system or method listed is relevant to your study. If you are not sure if a list item applies to your research, read the appropriate section before selecting a response.

Materials & experimental systems

n/a	Involved in the study	
☐	☒	Antibodies
☐	☒	Eukaryotic cell lines
☒	☐	Palaeontology and archaeology
☒	☐	Animals and other organisms
☒	☐	Clinical data
☒	☐	Dual use research of concern
☒	☐	Plants

Methods

n/a	Involved in the study	
☐	☒	ChIP-seq
☒	☐	Flow cytometry
☒	☐	MRI-based neuroimaging

Antibodies

Antibodies used	mNET-seq: RNA Polymerase II antibody, monoclonal (MBL Life science, CMA601, MABI0601, C13B9) (30 µg were used per 2e8 BLaER1 cells); ChIP-seq: Cyclin T1 antibody, monoclonal (Cell Signaling, 81464, clone D1B6G) (12.5 µl were used per 50 µg of BLaER1 chromatin), CDK9 antibody, monoclonal (Abcam, ab239364, clone EPR22956-37) (7.9 µg were used per 50 µg of BLaER1 chromatin), Drosophila H2Av antibody (Active Motif, 61686) (0.5 µg was used per 122 ng Drosophila spike-ins per 50 µg of BLaER1 chromatin); ChIP-nexus: RNA Polymerase II NTD antibody, monoclonal (Cell Signaling, 14958, clone D8L4Y) (12 µL were used per 60 µg of BLaER1 chromatin), Drosophila H2Av antibody (Active Motif, 61686) (1 µg was used per 244 ng Drosophila spike-ins per 60 µg of BLaER1 chromatin); Western Blotting: RNA Polymerase II antibody, monoclonal (Santa-cruz, sc-55492, clone F-12) (used in 1:200 dilution), GAPDH antibody, monoclonal (Sigma-Aldrich, G8795, clone GAPDH-71.1) (used in 1:20,000 dilution), Goat Anti-Mouse IgG - H&L (HRP) antibody, polyclonal (Abcam, ab5870) (used in 1:3,000 dilution).
Validation	Validation of the antibodies was performed by the manufacturer. The information provided below is taken from the websites of the corresponding manufacturers. RNA Polymerase II antibody (MBL Life science, CMA601, MABI0601, C13B9; RRID:AB_2827956) was validated by ELISA. Cyclin T1 antibody (Cell Signaling, 81464; RRID:AB_2799973) was validated by western blotting, immunoprecipitation, ChIP and CUT&RUN techniques. CDK9 antibody (Abcam, ab239364; RRID:AB_3096172) was validated by ChIP-seq, ChIC/CUT&RUN, western blotting, immunoprecipitation, immunohistochemistry, ChIP-qPCR, immunocytochemistry/immunofluorescence and flow cytometry techniques. Drosophila H2Av antibody (Active Motif, 61686; RRID:AB_2737370) was validated by ChIP-qPCR and ChIP-seq techniques. RNA Polymerase II NTD antibody (Cell Signaling, 14958; RRID:AB_2687876) was validated by western blotting, ChIP-qPCR and ChIP-seq techniques. RNA Polymerase II antibody (Santa-cruz, sc-55492; RRID:AB_630203) was validated by western blotting, immunoprecipitation, immunohistochemistry, immunofluorescence and ELISA techniques. GAPDH antibody (Sigma-Aldrich, G8795; RRID:AB_1078991) was validated by western blotting, immunocytochemistry, immunofluorescence, indirect ELISA and microarray techniques. Goat Anti-Mouse IgG - H&L (HRP) antibody (Abcam, ab5870; RRID:AB_955389) was validated by dot blotting, electron microscopy, immunohistochemistry, western blotting, ELISA and immunocytochemistry/immunofluorescence techniques.

Eukaryotic cell lines

Policy information about cell lines and Sex and Gender in Research

Cell line source(s)	The BLaER1 cell line was obtained from the laboratory of Thomas Graf (Rapino et al., Cell 2013). BLaER1 cells are a single subclone derived from C\EBPaER-GFP-transfected RCH-ACV B-cell leukemia cell line sorted for GFP expression (by the laboratory of Thomas Graf).
Authentication	Authentication was performed by FACS and transcriptome analysis by the laboratory of Thomas Graf (Rapino et al., 2013; Stick et al., 2020) and by our laboratory (Choi et al., 2021). STR profiling was performed by Millipore (see cat. # SCC165). Further information on BLaER1 authentication can be found in Cellosaurus, RRID:CVCL_VQ57.
Mycoplasma contamination	The BLaER1 cell line was regularly examined and tested negative for the mycoplasma contamination using Plasmo Test Mycoplasma Detection Kit (InvivoGen, rep-pt1).
Commonly misidentified lines (See ICLAC register)	No commonly misidentified cell lines were used.

Plants

Seed stocks	Not applicable
Novel plant genotypes	Not applicable
Authentication	Not applicable

ChIP-seq

Data deposition

☒ Confirm that both raw and final processed data have been deposited in a public database such as GEO.

☒ Confirm that you have deposited or provided access to graph files (e.g. BED files) for the called peaks.

Data access links *May remain private before publication.*	The sequencing data and processed files are deposited in the GEO database under accession code GSE235181.
Files in database submission	BLaER1_ChIPNexusseq_PolII_DMSO_30_0h_R1_S5_R2_001.minus.bw<br>BLaER1_ChIPNexusseq_PolII_DMSO_30_0h_R2_S6_R2_001.minus.bw

BLaER1_ChIPNexusseq_PolII_DMSO_30_96h_R1_S1_R2_001.minus.bw
BLaER1_ChIPNexusseq_PolII_DMSO_30_96h_R2_S2_R2_001.minus.bw
BLaER1_ChIPNexusseq_PolII_DMSO_6_0h_R1_S1_R2_001.minus.bw
BLaER1_ChIPNexusseq_PolII_DMSO_6_0h_R2_S2_R2_001.minus.bw
BLaER1_ChIPNexusseq_PolII_DMSO_6_96h_R1_S1_R2_001.minus.bw
BLaER1_ChIPNexusseq_PolII_DMSO_6_96h_R2_S2_R2_001.minus.bw
BLaER1_ChIPNexusseq_PolII_TRP_30_0h_R1_S7_R2_001.minus.bw
BLaER1_ChIPNexusseq_PolII_TRP_30_0h_R2_S8_R2_001.minus.bw
BLaER1_ChIPNexusseq_PolII_TRP_30_96h_R1_S3_R2_001.minus.bw
BLaER1_ChIPNexusseq_PolII_TRP_30_96h_R2_S4_R2_001.minus.bw
BLaER1_ChIPNexusseq_PolII_TRP_6_0h_R1_S3_R2_001.minus.bw
BLaER1_ChIPNexusseq_PolII_TRP_6_0h_R2_S4_R2_001.minus.bw
BLaER1_ChIPNexusseq_PolII_TRP_6_96h_R1_S3_R2_001.minus.bw
BLaER1_ChIPNexusseq_PolII_TRP_6_96h_R2_S4_R2_001.minus.bw
BLaER1_ChIPNexusseq_PolII_DMSO_30_0h_R1_S5_R2_001.plus.bw
BLaER1_ChIPNexusseq_PolII_DMSO_30_0h_R2_S6_R2_001.plus.bw
BLaER1_ChIPNexusseq_PolII_DMSO_30_96h_R1_S1_R2_001.plus.bw
BLaER1_ChIPNexusseq_PolII_DMSO_30_96h_R2_S2_R2_001.plus.bw
BLaER1_ChIPNexusseq_PolII_DMSO_6_0h_R1_S1_R2_001.plus.bw
BLaER1_ChIPNexusseq_PolII_DMSO_6_0h_R2_S2_R2_001.plus.bw
BLaER1_ChIPNexusseq_PolII_DMSO_6_96h_R1_S1_R2_001.plus.bw
BLaER1_ChIPNexusseq_PolII_DMSO_6_96h_R2_S2_R2_001.plus.bw
BLaER1_ChIPNexusseq_PolII_TRP_30_0h_R1_S7_R2_001.plus.bw
BLaER1_ChIPNexusseq_PolII_TRP_30_0h_R2_S8_R2_001.plus.bw
BLaER1_ChIPNexusseq_PolII_TRP_30_96h_R1_S3_R2_001.plus.bw
BLaER1_ChIPNexusseq_PolII_TRP_30_96h_R2_S4_R2_001.plus.bw
BLaER1_ChIPNexusseq_PolII_TRP_6_0h_R1_S3_R2_001.plus.bw
BLaER1_ChIPNexusseq_PolII_TRP_6_0h_R2_S4_R2_001.plus.bw
BLaER1_ChIPNexusseq_PolII_TRP_6_96h_R1_S3_R2_001.plus.bw
BLaER1_ChIPNexusseq_PolII_TRP_6_96h_R2_S4_R2_001.plus.bw
BLaER1_ChIPseq_Input_CT1_CDK9_0h_Rep1.bw
BLaER1_ChIPseq_Input_CT1_CDK9_0h_Rep2.bw
BLaER1_ChIPseq_Input_CT1_CDK9_24h_Rep1.bw
BLaER1_ChIPseq_Input_CT1_CDK9_24h_Rep2.bw
BLaER1_ChIPseq_Input_CT1_CDK9_96h_Rep1.bw
BLaER1_ChIPseq_Input_CT1_CDK9_96h_Rep2.bw
BLaER1_ChIPseq_CDK9_0h_Rep1.bw
BLaER1_ChIPseq_CDK9_0h_Rep2.bw
BLaER1_ChIPseq_CDK9_24h_Rep1.bw
BLaER1_ChIPseq_CDK9_24h_Rep2.bw
BLaER1_ChIPseq_CDK9_96h_Rep1.bw
BLaER1_ChIPseq_CDK9_96h_Rep2.bw
BLaER1_ChIPseq_CT1_0h_Rep1.bw
BLaER1_ChIPseq_CT1_0h_Rep2.bw
BLaER1_ChIPseq_CT1_24h_Rep1.bw
BLaER1_ChIPseq_CT1_24h_Rep2.bw
BLaER1_ChIPseq_CT1_96h_Rep1.bw
BLaER1_ChIPseq_CT1_96h_Rep2.bw

Genome browser session
(e.g. UCSC)

Not applicable

Methodology

Replicates

ChIP-seq and ChIP-nexus experiments were performed in two independent biological replicates. Detailed correlations between replicates are shown in the Extended Data section of this study.

Sequencing depth

The samples were sequenced using NextSeq550 Illumina platform to a depth of 30-40 million reads per sample for ChIP-seq experiments and to a depth of 80-100 million reads per sample for ChIP-nexus experiments.

Antibodies

ChIP-seq: Cyclin T1 antibody (Cell Signaling, 81464), CDK9 antibody (Abcam, ab239364), Drosophila H2Av antibody (Active Motif, 61686); ChIP-nexus: RNA Polymerase II NTD antibody (Cell Signaling, 14958), Drosophila H2Av antibody (Active Motif, 61686).

Peak calling parameters

Peak calling was performed by MACS2 with default parameters.

Data quality

The quality of the data was assessed by comparing the samples to available P-TEFb ChIP-seq data (Caizzi et al., Molecular Cell 2021) and Pol II ChIP-nexus data (Shao et al., Nature Genetics 2017).

Software

Paired-end reads of 75 bp length were collected for the samples. Reads were quality checked using FastQC. Reads were mapped to the human genome (GRCh38) using the Bowtie2 aligner. Other details about processing are described in the Methods section of this study.
