# Supplementary material for: Promoter-proximal RNA polymerase II termination regulates transcription during human cell type transition
Source: Nat Struct Mol Biol. 2025 Feb 11;32(6):995–1005. doi: 10.1038/s41594-025-01486-9 (PMC12170340; doi:10.1038/s41594-025-01486-9)

Extended Data Fig. 4a

0 h

Marker (focused light)

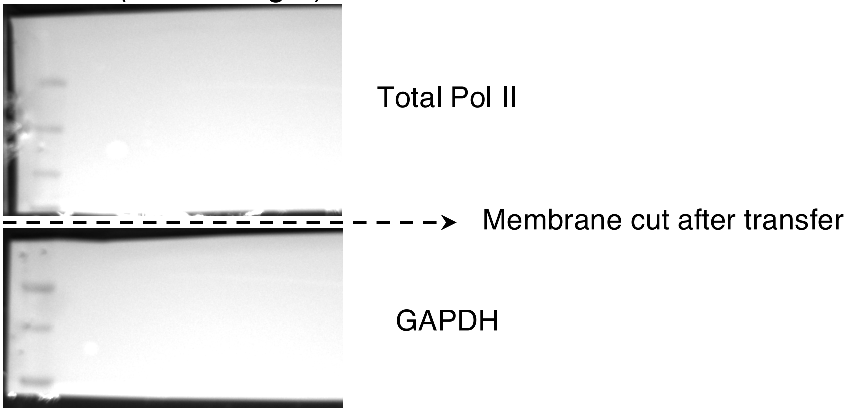

Signal detection (exposure time 10 sec)

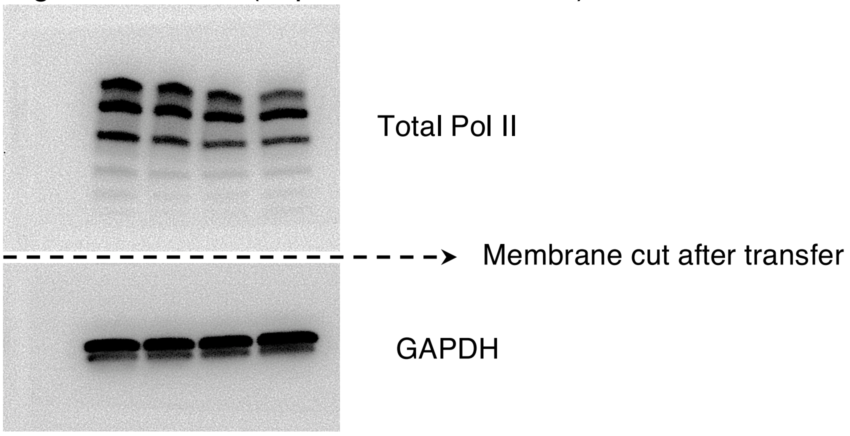

96 h

Marker (focused light)

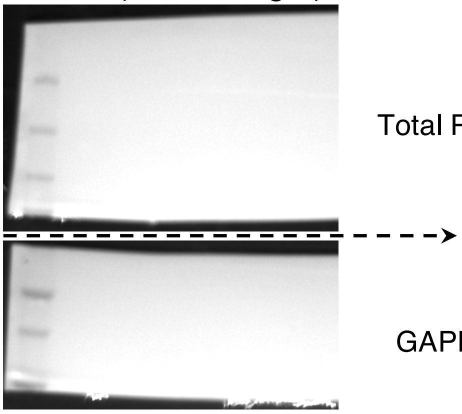

Signal detection (exposure time 10 sec)

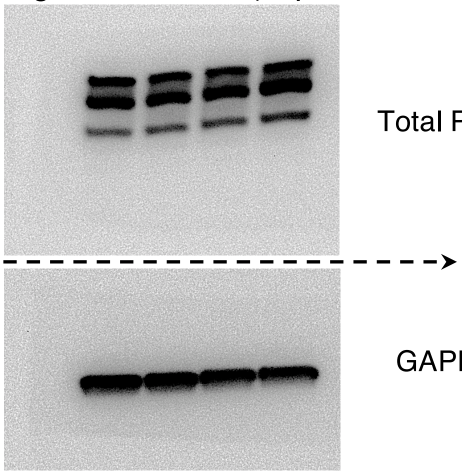

Supplement: Supplementary file 5 — Uncropped and unprocessed western blot scans. [file 41594_2025_1486_MOESM5_ESM.pdf]
